# Undergraduate medical teaching with remote consultations in general practice: a realist evaluation

**DOI:** 10.3399/BJGPO.2021.0185

**Published:** 2022-07-13

**Authors:** Roaa Al-bedaery, Umar Ahmed Riaz Chaudhry, Melvyn Jones, Lorraine Noble, Judith Ibison

**Affiliations:** 1 Institute of Medical & Biomedical Education, St George’s, University of London, Cranmer Terrace, London, UK; 2 Population Health Research Institute (PHRI), St George’s University of London (SGUL), University of London, Cranmer Terrace, London, UK; 3 UCL Medical School, Royal Free Hospital, London, UK

**Keywords:** education, medical, general practitioners, remote consultation, general practice, primary health care

## Abstract

**Background:**

Supervisors historically educated students in primary care in face-to-face contexts; as a result of COVID-19, students now experience patient consultations predominantly remotely. There is a paucity of evidence regarding the facilitators and barriers to supervising students for excellent educational impact in the remote consultation environment.

**Aim:**

To understand the facilitators and barriers to educating medical students using remote consultations in primary care, and the consequences for students in terms of educational impact.

**Design & setting:**

A realist evaluation methodology was adopted to identify causal chains of contexts, mechanisms, and outcomes, describing how the teaching and learning functioned on a sample of medical students and GP tutors from two medical schools in London, UK.

**Method:**

An initial programme theory was developed from the literature and a scoping exercise informed the data collection tools. Qualitative data were collected through online questionnaires (49 students, 19 tutors) and/or a semi-structured interview (eight students, two tutors). The data were coded to generate context–mechanisms–outcome configurations outlining how the teaching and learning operated.

**Results:**

The results demonstrated a sequential style of supervision can positively impact student engagement and confidence, and highlighted a need to address student preparation for remote patient examinations. Students found passive observation of remote patient encounters disengaging, and, in addition, reported isolation that impacted negatively on their experiences and perceptions of primary care.

**Conclusion:**

Student and tutor experiences may improve through considering the supervision style adopted by tutors, and through interventions to reduce student isolation and disengagement when using remote patient consultations in primary care.

## How this fits in

Remote consultations are increasingly used to teach medical students, but little is known about the facilitators and barriers to teaching and learning over this platform. This project explores the experiences of GP tutors and medical students with the use of remote consulting for undergraduate medical education during the COVID-19 pandemic. It highlights areas for improvement including supervision style, student engagement, and reducing student isolation.

## Introduction

The COVID-19 pandemic led to a surge in the use of remote consultations in primary care.^1^ This was followed by fast-tracked publications and guidance on how best to conduct these consultations,^2–4^ in what has been described as the fastest and most widespread innovation the NHS has experienced.^5^ This reduced medical students’ learning experiences with physically present patients,^6^ interrupting conventional methods of undergraduate teaching within primary care.

While the practicalities of remote consultations on the physician and patient experience have been evaluated,^7–9^ and are largely accepted, little is known about its value for medical student learning. Students have reported concerns with the reduced clinical exposure and variety of clinical experiences,^6,10^ as well as the impact this had on their skill acquisition and confidence. Therefore, the main research question was: what are the facilitators and barriers to GP tutors and medical students teaching and learning with remote (telephone and video) consultations in primary care? This realist evaluation aimed to explain what works, for whom, in what circumstances, and why. It is used here to describe the contextual drivers and inhibitors of student and tutor outcomes.

## Method

The realist evaluation utilises its explanatory power to understand why different outcomes occur,^11^ considering features of the learning environment (‘contexts’), how participants respond to these (‘mechanisms’), and what the consequences were in terms of the success of the learning activity (‘outcomes’).

### Study design

#### Developing the initial programme theory

The initial programme theory serves as a hypothetical representation of how the intervention (knowledge and skills transfer) is thought to function.^11^ This study used peer reviewed literature, practical experience, and a scoping exercise with key informants to guide and develop a set of anticipated context–mechanism–outcomes, as demonstrated in Supplementary Table S1.^6,12–28^

The study adopted mixed methods including an electronic survey and semi-structured interviews. The electronic survey was disseminated to medical students and GP tutors using an online survey tool (Jisc),^29^ across two medical schools in London, UK, between March and June 2021. Consenting participants were invited for interview between April and July 2021.

#### Questionnaire and semi-structured interview development

The questionnaire aimed to identify how GP tutors use remote consulting to supervise students, and offered opportunities for qualitative insights into the facilitators and barriers through open questions. The semi-structured interviews aimed to explore experiences and the meaning participants bring to these. In a realist evaluation, both sets of data are used to test the initial programme theory.^11^

The themes from the initial programme theory were used to construct the survey questions, using guiding principles.^30^ The student survey included 20 questions exploring the following: demographics, knowledge and skills, interaction with the remote patient, learning with remote consultations, and interaction with their supervisor. The tutor survey included 15 questions exploring the following: demographics, knowledge and skills, attitudes and attributes, and experience. These were explored in comparison with their experiences of teaching in face-to-face consultations with patients.

The semi-structured interview guides were developed using themes from the initial programme theory as well as iterative preliminary data from the study. The questions focused on feelings, perceptions, and experiences unpicking how different contexts drive various outcomes through different mechanisms.

Both the questionnaires and interview guides were tested on three representative participants of medical students, and three tutors for expert validation promoting methodological rigour.^31^ The surveys and semi-structured interview guides are provided in Supplementary Appendices A and B.

### Study setting and participants

Participants included medical students and GP tutors who have experienced teaching or learning with remote consultations in primary care from University College London (UCL) and St George’s University London (SGUL). Participants included final-year medical students at UCL, and both first clinical-year and final-year medical students from SGUL.

### Recruitment and data collection

Medical students were invited to complete the survey through forum advertisements and announcements following teaching sessions. GP tutors were contacted via email through administrative staff. The interviews were conducted via MS Teams by RA, lasting 30–45 minutes. These were audio-recorded, transcribed by a professional service, and anonymised.

### Data analysis

The data was uploaded to NVivo (version 12) and codes were generated to represent the initial programme theory. The data were iteratively read, and findings were categorised into contexts, mechanisms, and outcomes. The initial programme theory was amended iteratively based on emergent findings, allowing new data to be presented to participants during the interviews. Rationales for initial programme theory amendments were documented using memos and validated in discussion with senior colleagues. The use of NVivo was adapted from a realist perspective.^32^

## Results

### Participant demographics

Sixty-eight participants responded to the questionnaires (49 students and 19 tutors). The demographic details are summarised for students (Table 1) and tutors (Table 2). From these responders, eight students and two tutors completed an interview.

**Table 1.** Characteristics of the student research survey participants (n = 49)

Characteristic	Students, *n* (%)
**Institution**	
UCL	10 (20)
SGUL	39 (80)
**Sex**	
Male	20 (41)
Female	27 (55)
Prefer not to say	2 (4)
**Year of study**	
First clinical year	18 (37)
Second clinical year	2 (4)
Final year	29 (59)

SGUL = St George's University London. UCL = University College London.

**Table 2.** Characteristics of the tutor research survey participants (*n* = 19)

Characteristic	Tutors*, n* (%)
**Institution**	
UCL	16 (84)
SGUL	3 (16)
**Sex**	
Male	6 (32)
Female	13 (68)
**Years of experience as a tutor**	
≤2 years	6 (32)
3–5 years	4 (21)
≥6 years	9 (47)

SGUL = St George's University London. UCL = University College London.

### Contexts, mechanisms, and outcomes

The contexts, mechanisms, and outcomes from the interviews and questionnaires are presented in Table 3 and described in detail below. Additional data from the questionnaires are summarised in Supplementary Tables S2 and S3, and Appendix C.

**Table 3.** The context–mechanism–outcome configurations for the study

	Configuration	Context	Mechanism	Outcome
**Engagement**	1	Tutor offers opportunities for student to practise remote consultations	Student engagement through participationReduced student apprehension with remote consulting	Increased student confidence with remote patient consultationsImproved knowledge and skills
	2	Observational learning with remote consultationLimited opportunity to practise remote consultations	DisengagementDifficulty establishing tutor-student rapport	Dissatisfaction with learning opportunityReduced feedback from tutor to student
**Isolation**	3	Lack of team interactionLack of acknowledgement	Feeling of student isolationLack of patient physical examinations	Negative perception of general practice
**Preparation**	4	Inadequate student training for examining remote patients	Uncertainty with remote patient examinations	Lack of confidence with examining patients remotely
	5	Appropriate patient selection avoiding follow-ups, medication reviews, and patients with language barriers	Team organisation	Effective teaching
**Supervision**	6	Tutor offers opportunities for student to practise remote consultationsThe absence of the patient	Student has more time to prepare	Increased quantity and quality of tutor feedback
	7	Sequential supervision styleFinal-year medical students	Student engagementLess student pressure when not directly observedStudent independence	Time consuming for tutorsPromotes confidence, and patient rapport
	8	Parallel supervision style	Student anxietyDisrupted consultation	Reduced patient rapportTime effective for tutors
**Skills**	9	Lack of physical patient examinations	Difficulty establishing rapport with patient	Reduced skill acquisition
	10	Technological setbacks with video consulting	Disrupted consultation	Reduced use of video consultingLittle or no exposure to video consulting skills
	11	Lack of non-verbal cues	Less memorable experience	Reduced reflective practice

### Engagement

In the context of students being given the opportunity to practise remote consulting, outcomes of increased student confidence and knowledge were reported. This was primarily through the mechanism of increased student engagement:

*'This allows students to have the opportunity of speaking to patients by themselves — it helps build confidence.'* (First clinical-year student questionnaire, SGUL)

*'I spent at least 50% of my time doing my own consultations, which I’m sure is where I learnt the most.'* (Final-year student interview, SGUL)

Conversely, those only exposed to observational opportunities reported reduced feedback, disengagement, a poor supervisory relationship, and dissatisfaction with the learning opportunity:

*'Not all tutors were comfortable allowing me to consult on my own, which hugely limited my learning. In these cases, I would watch them consult on the phone, which was a very limited learning experience.'* (Final-year student questionnaire, SGUL)

*'Hard to contribute to the consultation when done remotely even on speakerphone. I found it easy to participate during F2F* [face-to-face] *without interfering with the flow.'* (First clinical-year student questionnaire, SGUL)

*'Feels like a waste of time. Very easy to zone out when you are not involved in the conversation and the patient isn't sat there in front of you. Not engaging at all.'* (Final-year student questionnaire, SGUL)

This disengagement with observing remote consultations was mitigated in the context of tutors actively involving students in the consultations:

*'… he would tell the patient that he was just going to pause for a second. So, he would mute the microphone and turn to me and ask me what I thought, to try and include me and be “oh, is there any questions that you think I should ask, or anything that you think I’ve missed.” So, very inclusive like that, which I thought really helped … '* (First clinical-year student interview, SGUL)

While the initial programme theory predicted students may feel apprehensive about remote consulting, the students felt more at ease when given the opportunity, compared with face-to-face consultations:

*'Adopt a very relaxed posture, sip your drink etc — can't do any of that if a patient is in the room.'* (Final-year student questionnaire, SGUL)

*“It* [the consultation] *becomes less stressful. You don’t have to sort of control your facial expressions.'* (Final-year student interview, SGUL)

### Isolation

An unpredicted emergent mechanism from the interviews was isolation, where seven out of eight students reported feeling left out and not part of the team. This led to a negative change in student perception of general practice (*n* = 15) following their experience with predominant remote consulting, reporting a reduced desire to pursue it as a career:

*'The pandemic made it a lot more lonely because there was no communal area to eat lunch or sit and have a tea, which is what I had on previous placements. And I think that’s also, it seems like that’s more of the reality of a GP anyway because you spend your lunch doing paperwork. But that, I felt the loneliness more because of the pandemic.'* (Final clinical-year student interview, UCL)

*'I felt like sort of an outsider at the practice even though I was there for five weeks because I wasn’t there for very long each day. I didn’t really feel like part of the team.'* (First clinical-year student interview, SGUL)

*'I was quite interested in GP* [general practice] *before this rotation, I was sort of between GP and psychiatry. I was sort of asking them, you know, “Do you think it will stay like this? Do you think it will go back to the way it was?” And they were basically saying, they think it will stay as it is now because it’s so much more efficient. And I just, I don’t know, the thing I like about medicine is meeting people and, you know, interacting with people, and I sort of struggled to get that same level of interaction over the phone in terms of kind of social gratification.'* (Final-year student interview, UCL)

### Preparation

While most students had some form of preparation for remote consulting (*n* = 35), there was a desire for focused and simulated sessions addressing remote patient examinations:

*'I remember there were certain things that, and I’m thinking of when somebody who called up with aching joints in their hands or something. The GP was sort of asking them to squeeze their own hand as a bit of a kind of screening test, which was not something I’d seen done before and yes, I thought that, yes, something like that could be potentially helpful.'* (Final-year student interview, SGUL)

In agreement with the initial programme theory, students who reported exposure to a variety of patients associated this with a well-organised reception staff and GP tutor:

*'I think the receptionists were pretty good, actually, sort of assigning me a bit of a mix.'* (Final-year student interview, SGUL)

Students who experienced a lack of variety of clinical cases remotely (*n* = 9) reported that this contributed to remote patient consulting being an ineffective teaching tool for them. Negative experiences with patient selection included those with language barriers, those who required direct visual assessments, and patients requiring follow-ups or medication reviews:

*'It also made it difficult when people for who perhaps English wasn’t their first language and/or they were quite sort of poor historians, and those times, it felt like it would have been easier had they been there in front of me.'* (Final-year student interview, SGUL)

GP tutors overall attempted to organise patients with new problems or acute presentations, avoiding complex mental health and those with language barriers:

*'I choose patients who I know and can rely on being open to questioning. Tricky patients* [for teaching] *are those who do not speak English.'* (UCL tutor questionnaire)

### Supervision

In the context of students being given the opportunity to practise remote consulting, an outcome of improved frequency and quality of tutor feedback was reported compared with face-to-face consultations. This was largely from final-year students, who put this down to better time management and the absence of the patient:

*'I would say I received more feedback from my remote consultations as there was more time to present to the doctor and discuss differentials without the patient in the room/waiting around in a clinic room and we could call them back at our leisure.'* (Final-year student questionnaire, SGUL)

*'The tutor can give more honest feedback without worrying about how patient reacts.'* (Final-year student questionnaire, SGUL)

Students who were given minimal or no opportunities to consult with patients reported a negative supervisory relationship and subsequent feedback:

*'Very passive learning so no opportunity for feedback.'* (First clinical-year student questionnaire, SGUL)

This difference in feedback with remote consulting was shared too by tutors, who reported a more doctor-focused approach was adopted. Students also reported that the context of an absent patient in the room allowed them to look up guidance and be better prepared for the consultation. This too helped with the outcome of more focused feedback.

#### Structure of supervision

While a negative perception of remote patient consultations for patient care and education was predicted in the initial programme theory, this was not supported in the data. However, the structure of supervision did emerge within the final context, mechanisms, and outcomes. There appeared to be three main supervision styles with remote consulting, namely: sequential, parallel, and observational. A sequential style involved the student independently undertaking a remote consultation in the absence of their supervisor, and subsequently presenting the case. A parallel style involved the student undertaking a remote consultation in the physical presence of their supervisor. Observation involved the students observing a GP conduct remote consultations.

The sequential style of supervision appeared to be the preferred style in the context of final-year medical students, promoting independence and leading to outcomes of increased confidence and patient rapport:

*'I like this best as although it’s a bit scary at first it forces you to have a go and get a bit more confident. I like being able to say that I'm going to ask questions and then the GP will call them back to discuss management etc as it makes me feel less pressured as I'm taking the history.'* (Final-year student questionnaire, UCL)

*'Very useful in developing confidence and independence. Able to formulate our own management plans and discuss these with supervisors before calling back.'* (First clinical-year student questionnaire, SGUL)

Tutors also acknowledged this style took the pressure off students, although some tutors felt this style was too time-consuming:

*'I usually go to another room to take the pressure off the student; they really appreciate the space.'* (UCL tutor questionnaire)

In the context of parallel supervision, students were given feedback on their consultation style. This was associated with increased anxiety and disruption to the consultation, and led to an outcome of reduced student rapport with the patient. First clinical-year students, however, expressed that this style offered the support they required early in their training. Tutors acknowledged this style is more time effective:

*'Can be nerve-wracking having someone listening or watching over you. Sometimes I lose my train of thought or forget key things!'* (Final-year student questionnaire, UCL)

*'Saves time as tutor is able to supervise and/or intervene as required.'* (UCL tutor questionnaire)

### Skills

All students expressed concerns with the lack of patient examinations, which reduced their ability to establish rapport with patients. This negatively impacted their perceived skill acquisition and opportunistic learning opportunities; 80% of students reported they felt they were missing out on key learning opportunities by undertaking predominantly remote consultations. Tutors also shared this concern and negative impact on skill acquisition:

*'I can’t even put a face to a name, very difficult to build rapport with patient.'* (First clinical-year student questionnaire, SGUL)

*'If there was a woman presenting with a sore throat, but she had examination findings unrelated e.g. a stoma, then I wouldn’t be able to learn as much over the phone.'* (Final-year student questionnaire, SGUL)

*'I barely examined any patients … I think remote consultations take away from physical examinations, which need to be practiced and refined.'* (Final-year student questionnaire, SGUL)

*'They are not examining enough normal organs to recognise the abnormal.'* (UCL tutor questionnaire)

Technological setbacks were described solely in the context of video consulting. Its use was limited owing to connection problems and a perception that telephone consulting was adequate. This view was shared among students and tutors, except for the use of video consultations for paediatric patients:

*'Also, with like paediatric conditions, if they're moving around, if they're looking quite happy then that’s reassuring. So, I used videos for those kinds of consultations but sometimes it wasn’t necessary.'* (Final-year student interview, UCL)

Students found their remote consultations to be less impactful, and less memorable. In contrast to the initial programme theory, they in turn reported less reflection following remote cases and a reduced learning impact beyond the consultation itself. This was put down to a lack of visual cues, which students reported usually helped make patients more memorable:

*'Less reflection because there are less queues* [cues] *to reflect on e.g., body language, facial expression. Telephone consults rely heavily on intonation and pauses.'* (Final-year student questionnaire, UCL)

*'Strangely feels less real when it’s online and remote.'* (First clinical-year student questionnaire, SGUL)

*'You don't physically see the patient so it’s more difficult to fix them in your mind.'* (First clinical-year student questionnaire, SGUL)

## Discussion

### Summary

This study has provided insights into the facilitators and barriers of using remote patient consultations to teach and supervise students in primary care, from the perspective of students and tutors from two London medical schools. The contexts, mechanisms, and outcomes generated described the importance of active student participation and how this, in turn, engaged students, increasing their confidence and skill acquisition. Students who actively participated in conducting their own consultations also reported a positive learning experience and increased confidence. This participation in the patient’s journey, supported by tutors, contributed to the student’s sense of belonging. Students, particularly those in their final year, favoured the sequential supervisory style, which allowed them to practice independently. Students also reported that the impression the patient left on them was reduced with remote consulting and less impactful, leading to less memorable encounters, as well as less reflection and learning beyond the consultation itself.

### Strengths and limitations

A strength of the study included the adoption of a realist enquiry, which helped to inform the decisions made in the study, including the development of the initial programme theory, data collection, and analysis, which promoted methodological rigour. The involvement of tutors and students in the development of the data collection tools helped to rephrase any unclear statements and ensured the intended constructs were being measured, improving the validity of the data collection tools. Furthermore, the iterative nature of the study allowed the emergent findings to be presented to the participants for testing, helping to keep the views and experiences of the students and tutors at the forefront.

While relevance and rigour were sought from a smaller sample size,^33^ this may not have covered all the influential contexts, mechanisms, and outcomes at play, and a low uptake of tutors for interview is a noted limitation. Participants were recruited from two institutions and their views and experiences may not be generalisable to the contexts of others. There is also a notable difference in the proportion of students and tutors across the institutions. This imbalance is likely owing to differences in the respective acceptable recruitment channels. Furthermore, the chains of causality derived with a context–mechanism–outcome configuration may well, in fact, create the context for another chain. These create complex connections that may not always be as simple and situated as a context–mechanism–outcome configuration.

### Comparison with existing literature

A key finding in this study has been the importance of active student participation; this resonates with Dornan *et al* and Hay *et al,* who also described the importance of participation as a key driver in maximising student learning outcomes.^34,35^ Learner participation in the workplace can increase engagement and confidence, contributing to the student’s sense of belonging. In the present study, students who were encouraged to participate in remote patient encounters felt more included.

By contrast, a concerning outcome of this study was student isolation, which impacted negatively on their learning experience and perception of general practice. This included the lack of contact with the team and passive learning experiences, which is in keeping with findings by Seabrook,^36^ who found a key barrier to participation is a lack of legitimacy. Those students in the present study who reported isolation also described a lack of participation. The lack of engaging opportunities may have led to them not feeling like a legitimate part of the general practice team. Lave and Wenger’s learning theory concerning ‘communities of practice’^37^ acknowledges the social nature of learning: when the learner is actively participating with members of the community (for example, a GP tutor), they bring more meaning to experiences, resulting in transformative learning from knowledge to personal and memorable learning encounters. The findings of this study (for example, the lack of ‘educational impact’ of remote consultations), are congruent with this theory of learning. This can be seen in students who were not made part of the ‘community of practice’ (through passive or non-inclusive encounters) reporting feeling isolated and having poor learning experiences.

Goal setting during learning encounters can impact student learning outcomes positively.^38^ This was evidenced with examples of tutors engaging students during observation (for example, asking the student to contribute to history taking), and students describing positive experiences through being able to interrupt the consultation to allow teaching (for example, muting the microphone and asking the student to contribute).

Students in their final year favoured the sequential supervisory style, which allowed them to practise independently. The literature describes this as a ‘minimal supervision’ style,^39^ offering students encounters that closely resemble what they will do as a doctor, and thus facilitating the development of clinical reasoning skills. The student preference for this style is aligned with social learning theory as it legitimises the role of a student in the patient’s journey,^37^ and as part of the clinical team. Furthermore, positive students' perceptions of this style is identified as a result of the autonomy it confers.^39^ This study, however, is the first to explore this in the context of primary care and with remote patients. However, it is a style that requires relatively more time and a spare consulting room,^40^ acting as an inhibitor for tutors. In order for independent student consultations to be appropriate, this method requires detailed appraisal by the supervisor of the student’s learning needs. Tutors have reported this style to be enjoyable, feasible, and educationally beneficial.

Overall, there is limited evidence describing the experiences of students and tutors who use remote patient consultations in undergraduate medical education. Darnton *et al* reported a pilot intervention allowing 35 medical students to consult with patients remotely (from home) during their primary care placements, supervised remotely by their GP tutor.^41^ The results echoed the findings of this study, finding remote consultations an acceptable modality for learning dependent on various similar facilitators and inhibitors, including appropriate patient selection, technology, the environment, and the supervisory role.

### Implications for research and practice

Institutions can reflect on the contexts, mechanisms, and outcomes identified in this study to better understand and deliver teaching with remote patient consultations. Importantly, the study has highlighted potential avenues to improve experiences and perceptions of medical students in general practice placements, including interventions to address student isolation and disengagement. Student isolation was associated with reduced social interaction, acknowledgement, and patient examinations, which can be explored further in future research. Tutors can consider their supervision style and encourage active participation in remote consultations. This may include meaningful engagement and goal setting when they are being observed by medical students. Importantly, they can also consider how they make students feel included as part of the team, not only through active participation, but also with acknowledgement and integrative practice. The findings also highlight a greater need to prepare students for remote patient examinations, a deficiency that could also be considered by tutors and institutes.

In conclusion, divergent historic pressures — for example, patient convenience, economic efficiency, and now COVID-19 — have led to the inexorable rise of remote consultations. With the expansion of telehealth across primary care, the impact this will have on undergraduate medical education cannot be neglected. While students, patients, and tutors yearn for more face-to-face time in clinic, remote consultations are the reality of the present, and perhaps the future of outpatient medicine. While the essence of the clinical interaction remains, there are a number of barriers to this being used in the same way as face-to-face consultations, inviting adaptations to the remote platform for both students and tutors.

This study has explored teaching and learning with remote consultations in primary care. The use of a realist evaluation has highlighted the facilitators and barriers at play for both tutors and students. The study highlights a student preference for sequential supervision and identifies the drivers of disengagement, as seen with observational learning opportunities. Student isolation emerged as a key theme, driving student dissatisfaction and negative perceptions of general practice. Students need to be prepared for and effective at remote consultations for the future workforce. This study helps to target this need by offering institutions and tutors practical insights regarding improving student experiences and skills with remote consulting through, for example, task-oriented observational learning. It also offers potential avenues for future research, such as interventions to target disengagement.
